# Dual PARACEST and ^19^F MR molecular imaging of fibrin clots with targeted perfluorocarbon nanoparticles

**DOI:** 10.1186/1532-429X-11-S1-T6

**Published:** 2009-01-28

**Authors:** Kejia Cai, Lei Zhang, Jacob Myerson, Wenjing Huang, Shelton D Caruthers, Gregory M Lanza, Samuel A Wickline, Patrick M Winter

**Affiliations:** Washington University, School of Medicine, Saint Louis, MO USA

**Keywords:** Nanoparticle Concentration, Fibrin Clot, Chemical Exchange Saturation Transfer, Total Imaging Time, Increase Nanoparticle Concentration

## Introduction

Fibrin is an abundant component of thrombi and an early marker of ruptured atherosclerotic plaques, which are the major cause of myocardial ischemia and stroke. Identification of fibrin could help detect ruptured plaques and direct therapeutic interventions to prevent or ameliorate the consequences of a heart attack or stroke. Fibrin-targeted perfluorocarbon nanoparticles provide a unique platform for molecular imaging of thrombi by MRI. The particle surface can be formulated with MRI contrast agents, such as PARACEST (PARAmagnetic Chemical Exchange Saturation Transfer) chelates that can provide "contrast on demand" by simply turning on and off a prepulse. In addition, the perfluorocarbon core can be exploited for ^19^F imaging.^19^F offers high signal intensity and no background signal from biological tissues, yielding a unique signature.

## Purpose

To combine PARACEST and ^19^F imaging for the corroborative detection of thrombi and quantitating the binding of targeted nanoparticles.

## Methods

**Figure 1:**
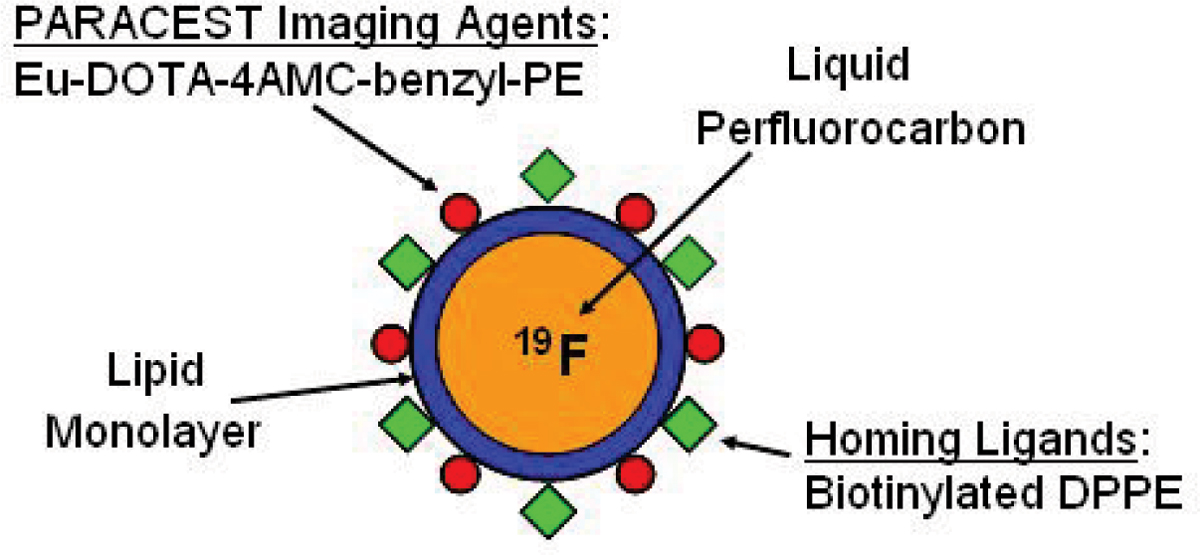
**Schematic representation of fibrin-targeted perfluorocarbon nanoparticles for MR molecular imaging**.

Fibrin-targeted PARACEST perfluorocarbon nanoparticles were prepared by incorporating a PARACEST chelate, Eu-DOTA-4AMC-benzyl-PE, and biotinylated dipalmitoylphosphatidylethanolamine (DPPE) into the lipid monolayer surface (Figure 1). A phantom was prepared by diluting the particles to 0.5, 1, 2, 4, 8 and 17 nM. Fibrin clots were prepared with dog plasma and suspended in normal saline. The targeted clots (n = 8) were treated with serial incubation of biotinylated anti-fibrin antibodies, avidin and biotinylated nanoparticles. Control clots (n = 5) were not incubated with nanoparticles. The phantom and clots were imaged with a 11.7 T horizontal bore scanner using a single turn solenoid coil that can be manually tuned to 500 MHz for PARACEST imaging or 470 MHz for ^19^F imaging. PARACEST images were obtained with a 2 second presaturation RF pulse (6.6 μT) applied at frequency offsets of ± 51 ppm at a resolution of 156 μm by 156 μm by 4 mm, with a 2.01 second TR, 2.5 millisecond TE and 8 signal averages, yielding a total imaging time of 34 minutes. ^19^F imaging was performed with identical settings except 1 second TR, 9 millisecond TE, 64 signal averages and 2 times of image matrix interpolation. PARACEST contrast to noise ratio (CNR) and the ^19^F signal to noise ratio (SNR) were calculated by manual selection of regions of interest.

## Results

**Figure 2:**
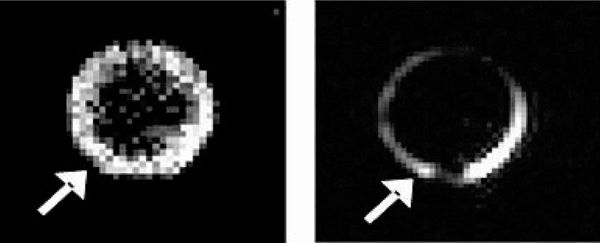
**PARACEST and** ^**19**^**F MR imaging of a clot (Arrow) treated with fibrin-targeted nanoparticles**.

The phantom images showed a linear increase in both PARACEST CNR and the ^19^F SNR with increasing nanoparticle concentrations, indicating that ^19^F imaging can corroborate the PARACEST signal and quantitate nanoparticle number. The 0.5 nM dilution showed a PARACEST CNR of 8.2, exceeding the minimum limit of detection (CNR = 5). Clots treated with targeted agent showed PARACEST enhancement along the clot boundary, and no enhancement in the clot interior or the surrounding saline (Figure 2). Similarly, the ^19^F images showed signal only at the clot surface. The PARACEST CNR was 23.6 ± 5.5 for the targeted clots but only 2.1 ± 0.2 for control clots. The ^19^F SNR was 12.6 ± 2.3 for the targeted clots, but only 1.8 ± 0.2 for the control clots. The ^19^F signal suggests that the nanoparticle concentration on the clot surface was 2.5 nM. Interestingly, the phantom experiment predicts that 2.5 nM of nanoparticles would only produce a PARACEST CNR of 15.6, which indicates that binding the nanoparticles to a target actually improves the PARACEST mechanism. This could result from slowing of the water exchange rate with the PARACEST chelate.

## Conclusion

Dual PARACEST and ^19^F MR molecular imaging of fibrin with targeted PARACEST perfluorocarbon nanoparticles was demonstrated at 11.7 T. Phantoms showed the detection limit of the PARACEST nanoparticles was <500 pM. The PARACEST enhancement observed on the clot surface was higher than the expected value based on the ^19^F signal, suggesting that binding the nanoparticles to a target improves the efficacy of the contrast mechanism.

